# Experimental Liver Cirrhosis Inhibits Restenosis after Balloon Angioplasty

**DOI:** 10.3390/ijms241411351

**Published:** 2023-07-12

**Authors:** Mare Mechelinck, Marc Hein, Carolin Kupp, Till Braunschweig, Marius J. Helmedag, Axel Klinkenberg, Moriz A. Habigt, Uwe Klinge, René H. Tolba, Moritz Uhlig

**Affiliations:** 1 Department of Anesthesiology, Faculty of Medicine, RWTH Aachen University, 52074 Aachen, Germany; 2 Department of Pathology, Faculty of Medicine, RWTH Aachen University, 52074 Aachen, Germany; 3 Department of General, Visceral and Transplantation Surgery, Faculty of Medicine, RWTH Aachen University, 52074 Aachen, Germany; 4 Institute for Laboratory Animal Science and Experimental Surgery, Faculty of Medicine, RWTH Aachen University, 52074 Aachen, Germany

**Keywords:** balloon dilatation, bile duct ligation, carotid injury, cirrhosis, neointimal condensation, rats, vascular remodeling

## Abstract

The effect of liver cirrhosis on vascular remodeling in vivo remains unknown. Therefore, this study investigates the influence of cholestatic liver cirrhosis on carotid arterial remodeling. A total of 79 male Sprague Dawley rats underwent bile duct ligation (cirrhotic group) or sham surgery (control group) and 28 days later left carotid artery balloon dilatation; 3, 7, 14 and 28 days after balloon dilatation, the rats were euthanized and carotid arteries were harvested. Histological sections were planimetrized, cell counts determined, and systemic inflammatory parameters measured. Up to day 14 after balloon dilatation, both groups showed a comparable increase in neointima area and degree of stenosis. By day 28, however, both values were significantly lower in the cirrhotic group (% stenosis: 20 ± 8 vs. 42 ± 10, *p* = 0.010; neointimal area [mm^2^]: 0.064 ± 0.025 vs. 0.138 ± 0.025, *p* = 0.024). Simultaneously, cell density in the neointima (*p* = 0.034) and inflammatory parameters were significantly higher in cirrhotic rats. This study demonstrates that cholestatic liver cirrhosis in rats substantially increases neointimal cell consolidation between days 14 and 28. Thereby, consolidation proved important for the degree of stenosis. This may suggest that patients with cholestatic cirrhosis are at lower risk for restenosis after coronary intervention.

## 1. Introduction

Liver cirrhosis is a known risk factor for coronary artery disease (CAD) [1,2]. In addition, cirrhotic patients have been shown to have increased mortality and a higher risk of adverse events, such as myocardial infarction, following percutaneous coronary intervention (PCI) [3,4,5]. There even appears to be a correlation between the degree of liver fibrosis and the risk of cardiovascular and overall mortality following PCI. Therefore, liver fibrosis scores in coronary artery disease patients are increasingly proposed as general predictors of outcomes after PCI [6,7]. However, in spite of these findings, no hypothesis has yet been formed regarding the pathomechanisms, underlying the increased cardiovascular risks in cirrhotic patients. A first thought might be an increased hyperplasia of the neointima, due to an accelerated migration and proliferation of vascular smooth muscle cells (SMCs) [8]. However, other processes of vascular remodeling also influence the final degree of stenosis [9], such as vascular recoil [10] and condensation of the neointima [11]. The increased inflammatory state in patients with liver cirrhosis might enhance stenosing processes [12], whereas other factors such as elevated bilirubin [13] and bile acid levels [14] might inhibit them. Thus, the overall effect of liver cirrhosis on vascular healing after PCI in vivo remains elusive. We recently provided a first clue with an in vitro experiment: sera from patients with alcoholic liver cirrhosis inhibited the proliferation and migration of human coronary artery SMCs [15]. To date, however, it is not clear whether the same process alterations can also be observed in vivo.

This study, therefore, investigates for the first time the effects of chronic liver cirrhosis (cirrhotic group) compared to a control group on vascular remodeling after balloon angioplasty using an established model of carotid artery angioplasty in rats. Bile duct ligation (BDL) was used as a common animal model to induce advanced chronic liver disease in the cirrhotic group [16]. As a result, rats develop cholestatic liver disease and thereby cirrhosis of the liver. Cholestatic liver disease encompasses a group of heterogeneous diseases characterized by impaired bile flow within the liver and subsequent accumulation of bile acids, bilirubin and other cholestatic metabolites, as well as an increased inflammatory state. In advanced stages, it leads to cirrhosis of the liver. The two most common etiologies of cholestatic liver disease in humans are primary biliary cholangitis and primary sclerosing cholangitis [17].

## 2. Results

### 2.1. Pathology and Morphometry

**Figure 1:**
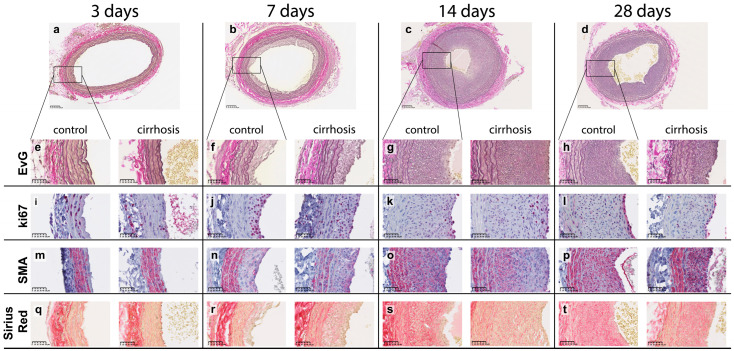
Representative histological cross-sections of the left common carotid artery 3, 7, 14 and 28 days after balloon dilatation in the control and cirrhotic group. Scale bars (in the lower left corner) correspond to (**a**–**d**) 100 µm and (**e**–**t**) 50 µm length.

Visual assessment of histologic sections revealed: the neointima area increased until day 14 and decreased thereafter (Figure 1). In both groups, the luminal cells proliferated more frequently (more ki67 positive (ki67+) cells). The proportion of smooth muscle actin (SMA) and Sirius Red (SR) positive areas within the neointima increased over time.

### 2.2. Neointima Area and Vascular Stenosis

**Figure 2:**
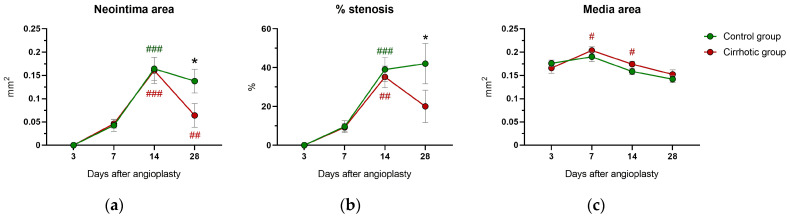
Results of planimetric analysis of histological cross-sectional areas of common carotid arteries in rats after bile duct ligation (cirrhotic group) versus sham surgery (control group) 3, 7, 14 and 28 days after vessel injury by balloon dilatation. Shown are (**a**) the size of the neointimal area, (**b**) the percentage of vascular stenosis and (**c**) the size of the media area. Values are presented as mean ± standard errors of the mean (SEM). *p*-values (from generalized linear mixed model and pairwise group comparisons corrected for multiple comparisons): * *p* < 0.05 control vs. cirrhosis; # *p* < 0.05 vs. previous time point in that group; ## *p* < 0.01 vs. previous time point in that group; ### *p* < 0.001 vs. previous time point in that group.

The size of the neointimal area (Figure 2a) as well as the relative vascular stenosis (Figure 2b) increased significantly in both groups until day 14 after vessel injury (day 7 to 14: *p* < 0.01). No overall differences were found between the groups in terms of neointimal area (*p*_group_ = 0.198) or relative vascular stenosis (*p*_group_ = 0.086). Between days 14 and 28 after vascular injury, the neointimal area decreased significantly in the cirrhotic group (*p* = 0.004) and the degree of stenosis declined, but not significantly (*p* = 0.145). In contrast, in the control group, there were only minor changes in relative vascular stenosis (*p* = 0.736) and neointima area (*p* = 0.302) in the same period. Consequently, 28 days after vascular injury, the neointima area (*p* = 0.024) and vascular stenosis (*p* = 0.010) were significantly lower in the liver cirrhosis group than in the control group. 

### 2.3. Media Area and Circumferences

The transient increase in media area from day 3 to 7 was followed by a decrease (Figure 2c). Both trends were significant only in the cirrhotic group (day 3 to 7: *p* = 0.019; day 7 to 14: *p* = 0.034). Nevertheless, there were no group differences in the media area (*p* = 0.118). The length of the external (EEL) and internal elastic laminae (IEL) demonstrated a comparable time course: at day 7, both circumferences were significantly longer in the cirrhotic than in the control group (IEL: *p* = 0.042 and EEL: *p* = 0.034, Supplementary Figure S1). The ratio of EEL to IEL does not differ between the groups (*p* = 0.865, Supplementary Figure S1).

### 2.4. Immunohistology and Immunohistochemistry

#### 2.4.1. Neointima

**Figure 3:**
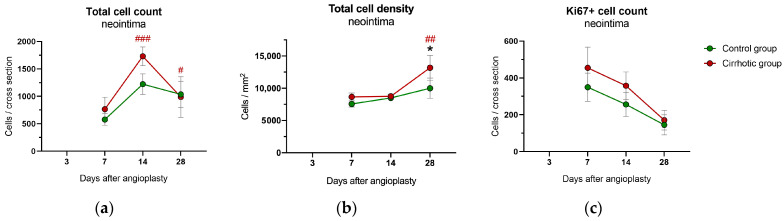
Immunohistological analysis of the neointima from the common carotid arteries of rats after bile duct ligation (cirrhotic group) versus sham surgery (control group) 3, 7, 14 and 28 days after balloon dilatation: (**a**) the overall cell count per vessel cross-section, (**b**) the general cell density and (**c**) the ki67 positive (ki67+) absolute cell count per vessel cross-section. Values are presented as mean ± standard errors of the mean (SEM). *p*-values (from generalized linear mixed model and pairwise group comparisons corrected for multiple comparisons): * *p* < 0.05 control vs. cirrhosis; # *p* < 0.05 vs. previous time point in that group; ## *p* < 0.01 vs. previous time point in that group; ### *p* < 0.001 vs. previous time point in that group.

In the cirrhotic group, the total cell count in the neointima significantly increased from day 7 to 14 (*p* < 0.001) and subsequently decreased until day 28 (day 14 to 28: *p* = 0.024) (Figure 3a). Compared to the control group, which showed a similar time course but without significant changes, no differences could be found. Cell density increased from day 14 to 28 only in the cirrhotic group (*p* = 0.007) and was thus significantly higher in the cirrhotic group on day 28 (*p* = 0.034) (Figure 3b). 

The number of ki67+ cells per cross-section in the neointima decreased from day 7 to 28 in both groups (*p*_time_ = 0.004) (Figure 3c). There were no significant group differences in the absolute count of ki67+ cells (*p*_group_ = 0.429) (Figure 3c), or their fraction of total cells (*p*_group_ = 0.481; Supplementary Figure S2).

The percentage of smooth muscle actin positive (SMA+) and Sirius Red positive (SR+) area increased continuously in the neointima between day 7 and day 28 in both groups (for SMA+ and SR+ both: *p*_time_ < 0.001), with no significant group differences (SMA+: *p*_group_ = 0.532; SR+: *p*_group_ = 0.138) (Supplementary Figure S2).

#### 2.4.2. Media

**Figure 4:**
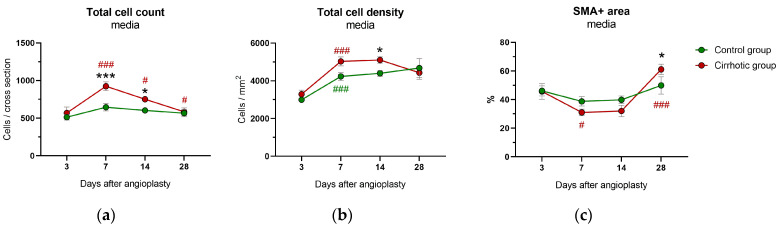
Immunohistological analysis of the media area of the common carotid arteries from rats after bile duct ligation (cirrhotic group) versus sham surgery (control group) at 3, 7, 14 and 28 days after balloon dilatation: (**a**) the overall cell count per vessel cross-section, (**b**) the total cell density and (**c**) the smooth muscle cell actin positive (SMA+) area are shown. Values are presented as mean ± standard errors of the mean (SEM). *p*-values (from generalized linear mixed model and pairwise group comparisons corrected for multiple comparisons): * *p* < 0.05 control vs. cirrhosis, *** *p* < 0.001 control vs. cirrhosis; # *p* < 0.05 vs. previous time points in that group; ### *p* < 0.001 vs. previous time points in that group.

Between day 3 and 7 after vascular injury, there was a transient significant increase in the total cell count in the media area of the cirrhotic group (*p* < 0.001), followed by a decrease until day 28 (day 7 to 14: *p* = 0.021; day 14 to 28: *p* = 0.043) (Figure 4a). The absolute cell count was significantly higher in the cirrhotic group on day 7 (*p* < 0.001) and 14 (*p* = 0.016), with a significantly higher cell density on day 14 (*p* = 0.022) (Figure 4b). After day 7, the cellular density did not change significantly.

The absolute number of ki67+ cells and the cell density of ki67+ cells in the media area decreased over time (number of ki67+ cells: *p*_time_ = 0.009; cell density of ki67+ cells: *p*_time_ = 0.012), with no significant differences between groups (number of ki67+ cells: *p*_group_ = 0.741; cell density of ki67+ cells: *p*_group_ = 0.319) (Supplementary Figure S3).

At 28 days after vascular injury, the relative SMA+ area was significantly higher in the cirrhotic group compared to the control group (*p* = 0.041) (Figure 4c). Whilst no significant changes over time were observed in the control group, the relative SMA+ area in the media of the cirrhotic group decreased significantly from day 3 to day 7 (*p* = 0.037) and increased again from day 14 to day 28 (*p* < 0.001). The relative SR+ area in the media remained stable over time (*p*_time_ = 0.058), with comparable values in both groups (*p*_group_ = 0.859) (Supplementary Figure S3).

### 2.5. Inflammatory Parameters and Liver Values Following Balloon Dilatation

**Figure 5:**
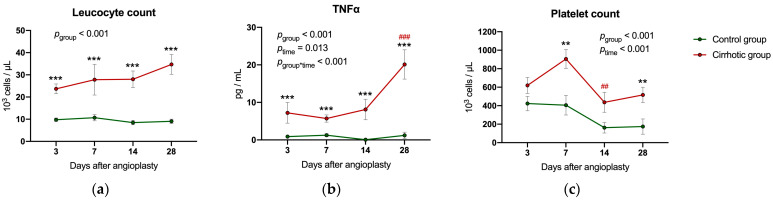
Laboratory diagnostics: the figure shows (**a**) the leucocyte count, (**b**) the level of tumor necrosis factor alpha (TNFα) and (**c**) the number of platelets 3, 7, 14 and 28 days after vascular injury in both experimental groups. Values are presented as mean ± standard errors of the mean (SEM). *p*-values (from generalized linear mixed model and pairwise group comparisons corrected for multiple comparisons): ** *p* < 0.01 control vs. cirrhosis; *** *p* < 0.001 control vs. cirrhosis; ## *p* < 0.01 vs. previous time points in that group; ### *p* < 0.001 vs. previous time points in that group.

The number of systemic leukocytes was significantly higher in the cirrhotic group compared to the control during the entire observational period (*p*_group_ < 0.001) (Figure 5a), while the number of platelets was significantly higher only on days 7 and 28 (*p*_group_ < 0.001) (Figure 5c). Tumor necrosis factor alpha (TNFα) increased continuously over time only within the cirrhotic group, but levels were consistently higher than in the control group (*p_group_* < 0.001) (Figure 5b).

Moreover, aspartate aminotransferase (AST), alkaline phosphatase (AP), creatinine and bilirubin levels were elevated and albumin and glucose levels were reduced in cirrhotic animals from day 3 (creatinine from day 7) to day 28 (Supplementary Table S1). 

## 3. Discussion

This study is the first to describe the effects of experimental liver cirrhosis on the pathological anatomy of vascular healing following carotid artery balloon dilatation in rats. While the proliferative phase up to day 14 remained unaffected by cirrhosis, an increased condensation of the neointima in the cirrhotic animals led to a lower degree of stenosis after 28 days. Elastic recoil did not account for the effect.

### 3.1. Proliferative and Migratory Phase until Day 14

In most studies, the process of neointimal formation is described as follows: subsequent to an initial inflammatory [8,18] or repair phase [19], neointimal hyperplasia occurs [18], which includes smooth muscle cell proliferation and migration [8,19] and increased extracellular matrix (ECM) deposition [20]. The time course varies depending on the species and type of vascular injury [21]. Most studies in rats show a maximal extent of the neointimal area at the end of their observational period, which is usually 28 days after vascular injury [22,23,24,25]. However, Han et al. describe no further increase in the neointima area between days 14 and 28 [26], similar to the control group in the present study. The proliferation rate of cells in the present study was highest in both the media and the neointima on days 3 and 7. This is consistent with previous findings [27,28]. In summary, the proliferative phase in this experiment ends around day 14. Up to this phase, there were no group differences in neointima due to cirrhosis (same neointima area, same cell number, same cell density, same number of proliferating cells and same SMA+ and SR+ area). Thus, experimental cirrhosis did not seem to affect neointimal development in the proliferative and migratory phases of this in vivo experiment. 

Only the media in the cirrhosis group showed a transient increase in cell count and density during the proliferative and migratory phase, which was accompanied by a symmetric increase in IEL and EEL. The increase in cells in the media has been described previously [23], but the transient positive remodeling (increase in IEL and EEL) has not been described before and its cause remains unclear. The timing of the transient circumferential change (it did not occur immediately after injury but was delayed) and the fact that there was no group difference in aortic compliance (Supplementary Figure S4) both suggest that this was not a passive, immediate response to balloon dilatation, i.e., no elastic recoil [29,30]. Rather, an active process must be assumed. Similar to the trend in the control group of the present study, Indolfi et al. described a slight but continuous decrease in IEL and EEL after balloon injury [22].

### 3.2. Neointimal Condensation 28 Days after Balloon Dilatation

The proliferative and migratory phase is followed by a phase of remodeling [8]. In our experiment, like in previous studies [30], the remodeling phase started between days 14 and 28. During this phase, the neointimal area decreased in the cirrhosis group by approximately 60% with a concomitant increase in neointimal cell density. Likewise, the degree of stenosis decreased in the cirrhotic group, but only by 40%, which can be attributed to negative remodeling (recognizable by the decline in IEL and EEL). Negative remodeling occurred to a similar extent in both groups and accounted for a decrease in lumen area of about 24 % in the observed period.

A decrease in the neointimal area over time has previously been described in some studies after balloon dilatation or stent implantation [30,31,32]. The cause of the decrease in neointima area has been attributed to a consolidation and regular arrangement of the cell layers [31] or degradation of the ECM by matrix metalloproteases [11,30], sometimes also reported as maturation of the ECM (degradation of proteoglycan elements and transition to elastin with some collagen) [32]. However, there are numerous other possible causes: for example, transforming growth factor beta 1 (TGFβ1), nitric oxide, oxidative stress, and SMC phenotype switching [9,30] are also involved in arterial remodeling. Various links with cirrhosis could be constructed here, but are not helpful at this stage. Instead, further studies are needed that focus on changes in neointima size and the degree of stenosis, also investigating possible causes. We could observe a trend towards a larger SMA+ and smaller SR+ area in the cirrhotic animals on day 28 (Supplementary Figure S2). Thus, the differentiation of neointimal cells and ECM restructuring could be targets for future investigations. 

### 3.3. Limitations

In this rat model, vascular healing was only studied in healthy, non-atherosclerotic rats. The advantage is certainly that it facilitates the discussion on the underlying causes. However, for better clinical transferability, an investigation into atherosclerotic vessels will be necessary in the future.

Although balloon dilatation of the rat common carotid artery (CCA) is the most commonly used model to study restenosis [33], it has a few limitations when transferred to humans: (i) the rat CCA is an elastic vessel, whereas the human coronary artery is a muscular vessel, so muscular effects cannot be represented in the model [33]. (ii) Rats are highly resistant to the formation of atherosclerosis [34]. (iii) Reendothelialization after vessel injury is significantly faster in rats than in humans [8], so the time course may not be transferable to humans. (iv) Only cholestatic cirrhosis can be assessed. Other forms of cirrhosis may differ substantially in the results. 

This study did not investigate the influence of possible collateral formation on vascular flow. Vascular endothelial growth factor (VEGF), which appears to be differentially regulated in cirrhosis depending on etiology and severity [35,36], has been shown to stimulate vessel growth [37] but also accelerates neointima formation in vascular remodeling [38]. Increased restenosis could thus be compensated by increased formation of collaterals. The effect on the formation of collaterals would have to be investigated separately in future studies.

### 3.4. Clinical Relevance

To the best of our knowledge, this study is the first to describe that cholestatic rats showed a pronounced neointimal condensation between 14 and 28 days after balloon dilatation. The observed effect is noteworthy. Transferred to the clinic, this could imply that patients with cholestatic liver cirrhosis might have a lower risk of restenosis after coronary intervention.

The reduced risk of restenosis is not what we initially expected given the proven elevated risk of patients with liver cirrhosis undergoing PCI [3,6,7,39,40]. However, there is evidence that the etiology of liver cirrhosis appears to have a strong and underestimated impact on cardiovascular risk [41,42]; for example, comparisons after liver transplantation have shown that cholestatic cirrhosis seems to be associated with a lower risk of cardiovascular events than nonalcoholic steatohepatitis (NASH) [43,44]. Likewise, the risk of developing CAD in the first place does not appear to be increased in cholestatic cirrhosis [45,46,47], whereas numerous studies have shown a significantly increased risk of CAD for NASH [41,47,48,49]. This, in turn, matches our results and would fit with the above-mentioned findings that bilirubin may have a protective effect on cardiovascular risk. In addition to bilirubin, elevated systemic bile acid levels in cholestatic cirrhosis may also play a protective role in CAD, as a prospective study in humans has shown that increased serum concentrations of bile acids were associated with a lower risk of CAD [14].

The results of this study highlight the need for similar studies investigating the effects of other etiologies of liver cirrhosis and raise the question of whether the neointimal condensation can be influenced therapeutically.

## 4. Materials and Methods

### 4.1. Animals and Housing

A total of 89 male Sprague Dawley rats (Rattus norvegicus; RjHan: SD; Janvier Labs, Le Genest Saint Isle, France) entered the experiment; 79 of 89 rats successfully completed the entire study protocol and were included in this study. Because of the high dropout rate (7 animals died prematurely and 3 animals were euthanized prematurely because of reaching a predefined humane end-point), the score parameters used were retrospectively evaluated and these results are published separately [50].

Apart from this, according to the 3Rs principle of Russel and Burch [51], this study was designed to investigate two distinct research aspects: the effect of liver cirrhosis on vascular remodeling after carotid artery damage (as discussed in this manuscript) and the effect of liver cirrhosis on cardiac function and structure [52].

The 79 rats weighed 494 ± 33 g (cirrhotic: 495 ± 34 g; control: 492 ± 33 g). Rats of this weight average about 12 weeks of age, according to the breeder (Janvier).

Rats were housed under specific pathogen-free conditions preferably in groups of two or three, at 22 °C, with 55% relative humidity, in rat filter top cages (Type 2000, Tecniplast, Hohenpreisenberg, Germany), with a 12:12 h light–dark cycle. Standard chow for laboratory rats (Ssniff GmbH, Soest, Germany) and sterile, acidified drinking water were provided ad libitum. Acclimatization period was at least 7 days before the start of the experiments.

### 4.2. Experimental Protocol

The experimental protocols were all approved by the public authority, the governmental animal care and use committee (No 84-02.04.2016.A391, Landesamt für Natur-, Umwelt- und Verbraucherschutz Nordrhein-Westfalen, Recklinghausen, Germany). All procedures were developed in accordance with The National Research Council Guide for the Care and Use of Laboratory Animals. The reporting in the manuscript adheres to the ARRIVE guidelines 2.0 [53].

**Figure 6:**
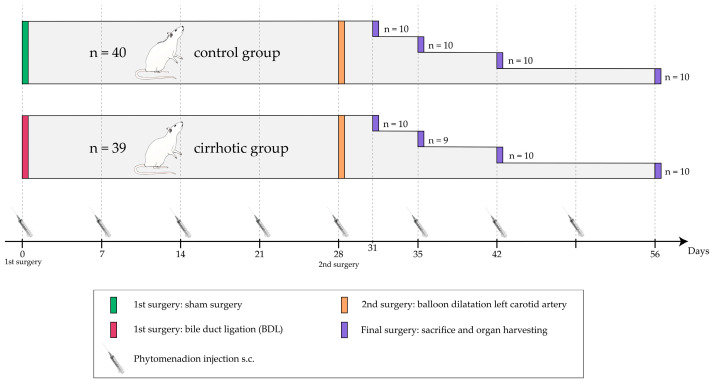
Experimental protocol: the timeline of the experiment is shown separately for the control and cirrhotic groups. Group sizes and time points (days after the first surgery) for each intervention are shown. This includes the first, second and final surgeries, as well as weekly subcutaneous phytomenadione injections.

Two consecutive surgeries were performed per rat (Figure 6); initially, animals underwent BDL or sham surgery to induce cholestatic liver cirrhosis in half of the rats. Four weeks later, vascular injury was induced by balloon dilatation of the left CCA in all rats. After another 3, 7, 14 or 28 days, 9 or 10 rats from each group were sacrificed and organs were harvested (Figure 6). The group assignment was done in a quasi-randomized manner. Blinding of the investigators was not possible since the BDL rats were visually distinguishable by their jaundice. All procedures were performed between 8:00 a.m. and 5:00 p.m. 

### 4.3. Anesthesia

Anesthesia was the same for each surgery: 30 min before induction of anesthesia, 0.01 mg/kg body weight (BW) buprenorphine (ESSEX PHARMA, Munich, Germany) was administered subcutaneously to the rats for analgesia. Subsequently, anesthesia was induced via 4 L of oxygen flow with 4 vol% isoflurane (Forene, 100% *v*/*v*, AbbVie, North Chicago, IL, USA) via an induction chamber and maintained using 2 L of oxygen flow with continuous 2 to 3 vol% isoflurane inhalation through a nose cone (HSE Anaesthesia Mask, 73-4861, Harvard Apparatus GmbH, Hugstetten, Germany). Rats remained spontaneously breathing throughout the procedures. During anesthesia, the following parameters were controlled for (1) heart rate from the electrocardiogram derived via needle electrodes; (2) peripheral oxygen saturation from pulse oximetry at the paw (Masimo Radical 7 Blue Screen, Irvine, CA, USA); and (3) body temperature from a rectal temperature probe. The temperature measurement was coupled to the heating pad via a feedback loop (TCAT-2LV controller, Physitemp, Clifton, NJ, USA) [54]. Depth of anesthesia was considered sufficient in the absence of a nociceptive response to the tail tip or interdigital pinch. Rats’ eyes were protected from dehydration using eye ointment (Bepanthen, Bayer, Leverkusen, Germany). Throughout the surgeries, any exposed tissue or organs were kept moist with a crystalloid solution. At wound closure, 25 mg/kg BW ropivacaine 0.5% (Ropivacaine Kabi 10 mg/mL, Fresenius, Bad Homburg v.d.H., Germany) was infiltrated locally. In addition, the animals received 100 mg/kg BW dipyrone subcutaneously (Novaminsulfon-ratio 1 g/2 mL, diluted to 100 mg/mL, Ratiopharm, Ulm, Germany) about 30 min before the end of surgery for postoperative pain therapy. This dipyrone administration was repeated once postoperatively as standard, and thereafter only as needed. The rats were then placed in a small animal intensive care unit with increased oxygen levels and warmth to recover from anesthesia (Vetario, Weston-super-Mare, UK). 

### 4.4. Cirrhosis Induction

After shaving and skin disinfection, a median laparotomy of approximately 2–3 cm was performed. The surgical field was kept open using small hooks. Using cotton swabs, the liver was carefully lifted and held cranially with a compress. For BDL the bile duct was cleared cranially from surrounding tissue, ligated twice with silk suture 5/0 (18020-50, Fine Science Tools, Vancouver, BC, Canada) and cut in between the ligations. The liver was carefully repositioned. As previously described, the sham surgery was performed in the same way as the BDL, except that the bile duct was not ligated and cut [55].

### 4.5. Carotid Artery Balloon Angioplasty

**Figure 7:**
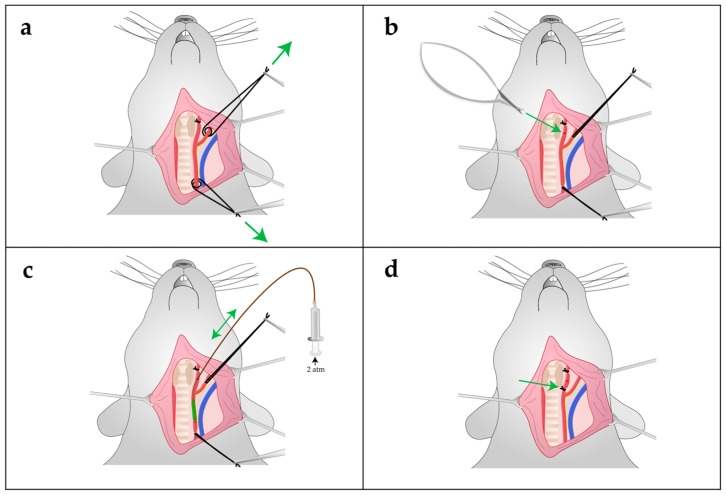
Schematic illustration of balloon dilatation of the left carotid artery (2nd surgery): showing a rat’s head and neck in dorsal recumbency. After medial incision of the skin and blunt preparation of the tissue, the vessels are visible (A. carotis [red], V. jugularis [blue]): (**a**) the external carotid artery was ligated cranially, and the internal and common carotid arteries (CCA) were double-looped and temporarily closed by traction (movement along the green arrows). (**b**) After transverse incision of the external carotid artery at the tip of the green arrow, (**c**) a balloon was advanced into the CCA, inflated and withdrawn three times with rotational movements (the green arrow indicates the forward and backward movement of the catheter). (**d**) The balloon was removed, and the external carotid artery ligated distal to the insertion site (at the tip of the green arrow). The temporary loops were then removed, and blood flow restored via the internal carotid artery.

Four weeks (28 days) after the first surgery, balloon dilatation of the left CCA was performed (Figure 7): the neck area was shaved and cleansed with skin disinfectant, followed by a median skin incision. All microsurgical steps were performed under a microscope. For the collection of 1.9 mL of blood and intravascular volume substitution, a central venous catheter (1-lumen catheter set, leaderflex 22G, VYGON GmbH & Co. KG, Écouen, France, 1212.04) was placed into the left external jugular vein after cranial ligature and transverse incision of the vessel. Access to the left CCA and arterial bifurcation was gained, and the vessel was separated from surrounding tissue. The external carotid artery was ligated cranially with silk suture 5/0 (Figure 7a). Moreover, silk sutures (5/0) were placed around the CCA and internal carotid artery to enable vascular intervention without bleeding (Figure 7a). A transverse arteriotomy of the external carotid artery distal to the ligature was performed with micro scissors (Figure 7b) and a 2-French Fogarty catheter was inserted and advanced through this vessel opening into the CCA (Figure 7c). The balloon was retracted three times and inflated with rotational movements from the aortic outlet up to the junction of the external carotid artery. After removing the balloon, the external carotid artery caudal to the incision was closed and blood flow via the CCA and internal carotid artery was restored (Figure 7d). 

### 4.6. Follow-Up Care

Throughout the whole experiment (4–8 weeks for each animal), the rats were visited at least daily. To avoid bleeding in liver cirrhosis, 2.5 mg/kg BW vitamin K (PZN 04273031, Phytomenadion, Konakion^®^ MM 10 mg/mL, F. Hoffmann-La Roche AG, Basel, Switzerland) was administered subcutaneously once a week in all animals.

### 4.7. Final Surgery: Sample Collection, Organ Harvesting and Sample Processing

Then, 3, 7, 14 or 28 days after balloon dilatation, the animals were anesthetized for the last time. Blood samples were taken. Under deep anesthesia, the animals were killed via removal of the heart. The carotid arteries were taken for histological processing. The tissue samples were fixed in formalin for 7 days, dehydrated through an ascending alcohol series and embedded in paraffin. The carotid arteries were cut twice to obtain 3 equal-sized sections before being embedded upright in paraffin. Sections of 3 µm thickness were cut from each of the paraffin blocks (carotid arteries in transverse) and mounted on microscope slides (StarFrost Advanced Adhesive, No. 11270, Engelbrecht GmbH, Edermünde, Germany). The slides were stained for hematoxylin and eosin and Elastica van Gieson (EvG) and scanned for further analysis. On average, 5 evaluable cross-sectional images per animal from different levels of the carotid artery were included in the analysis.

TNFα was measured in serum samples as a global inflammatory marker with a commercially available R&D ELISA kit (Rat TNF-alpha Quantikine ELISA Kit, RTA00, R&D Systems, Inc. Minneapolis, MN, USA) according to the manufacturer’s protocol. Analogous to the method M5 proposed by Beal et al. [56], values below the lower limit of detection (LoD) were replaced with LoD/2. Leukocyte and platelet counts were determined using an automated cell counter (Celltac Alpha VET MEK-6550, NIHON KOHDEN, Tokyo, Japan). To evaluate the degree of organ dysfunction, AST, AP, bilirubin, albumin and creatinine were measured by the Laboratory of Hematology at the Institute of Laboratory Animal Science and Experimental Surgery, RWTH Aachen University, Faculty of Medicine, Aachen, Germany. A blood gas analyzer (ABL800 FLEX, Radiometer, Copenhagen, Denmark) was used to determine the concentration of blood glucose.

### 4.8. Pathology and Morphometry

In the digitized EvG stained cross-sectional images of the CCA, the boundaries of the neointima and media area were manually traced by one blinded veterinarian in each section. The media and neointima area were then quantified using the image processing program Fiji (Fiji Is Just ImageJ, version 1.52s, Wayne Rasband, National Institute of Health, Bethesda, MD, USA) [57]. To measure the length of both the IEL and the EEL, in the images any fissures caused by tissue processing were closed manually. The aim was to match the actual circumferences of the neointima and media as closely as possible by excluding very small wrinkles in the IEL and EEL measurements, as described previously [58]. To avoid a bias due to manual smoothing in this process, the circumferences were smoothened in a standardized manner using Adobe Photoshop CC 2018 (Adobe Inc., San José, CA, USA). The measurements were performed automatically using Fiji. The area within the IEL (IEA) was calculated starting from the IEL, assuming a perfectly round vessel, as described previously [59,60]. The degree of stenosis was calculated as the ratio of the neointima area and IEA. For a description of the calculation of aortic compliance, see the description of Supplementary Figure S4.

### 4.9. Immunohistology and Immunohistochemistry

Carotid cross sections were stained for SR (Direct Red 80, Sigma-Aldrich Chemie GmbH, Taufkirchen, Germany; 365548), ki67 (with the antibody M7248 from Dako, Agilent Technologies, Santa Clara, CA, USA; 1:10 diluted) and SMA (M0851; Dako; 1:100 diluted). These sections were scanned. Subsequent analysis was conducted with QuPath software (version 0.2.3, Queen’s University, Belfast, UK) [61]: once more, the areas of media and neointima were first designated manually before the subsequent parameters were automatically quantified for these two areas. The positive cell detection command in QuPath was used for cell detection, counting and classification. In the ki67-stained sections, the overall cell number, as well as the cell density per mm² and the number of ki67+ cells per cross-section were determined. The threshold for positive cell detection was set using the pixel intensity histogram in order to ensure a standardized classification while still taking staining variation between slides into account. The formula used was:

threshold = mean pixel intensity + 8 × standard deviation of the pixel intensity.(1)

The relative proportion of SMA+ and SR+ areas was measured. For quantification of SMA-positive cells, it is common to analyze the stained areas [62,63], as a reliable assignment to individual cells is not possible.

### 4.10. Statistics

The needed sample size was calculated a priori using a power analysis: a weighted effect size (Hedge’s g 1.796) was calculated from data from two previous studies on the effect of diabetes or insulin on neointimal formation after 21 or 28 days [27,64]. Combined with a power of 0.95, an alpha error of 0.05 and an allocation ratio of 1, this resulted in 10 rats per group and the time point required to detect a significant difference between the groups. 

The statistical analysis was done using SPSS 29 (IBM Corporation, Armonk, NY, USA) and SAS/STAT 9.4 (SAS Institute, Cary, NC, USA). The graphs were created using GraphPad Prism 9 (GraphPad Software, San Diego, CA, USA). For single comparisons, an alpha level < 0.05 was considered statistically significant. Normal distribution was tested for using Shapiro–Wilk. For the platelets counts and TNFα values Box-Cox transformations were performed (platelets: lambda = 0.5; TNFα: lambda = 0). To analyze the results, generalized linear mixed models were performed with the group (cirrhosis vs. control), the time point (3, 7, 14 and 28 days after balloon dilatation of the carotid artery) and group × time point as fixed effects and an animal identification number as random effects. Results of multiple samples from one animal were included in the calculation as measurement replicates. Pairwise comparisons were added to compare the values of the two groups at the individual time points and between consecutive time points within each group. To correct for multiple comparisons in these tests, the significance level was adjusted using a sequential, stepwise Šidák procedure. For the pairwise comparisons, the adjusted *p*-values are reported. Mean values ± standard errors of the mean (SEM) are shown.

## 5. Conclusions

This study shows in a rat model of vascular healing after balloon dilatation of the CCA that cholestatic cirrhosis causes a substantial neointimal condensation and, therefore, approximately 52% less stenosis 28 days after vascular injury compared to control animals. These data demonstrate that, in addition to neointima formation and vascular remodeling, neointimal condensation is also relevant for the actual degree of stenosis. The results may be clinically important: they indicate that patients with cholestatic cirrhosis may have a lower risk of in-stent restenosis after PCI. The increased risk of mortality and adverse events after PCI in cirrhotic patients, as described in the literature, may therefore eventually be attributable to the non-cholestatic etiologies of cirrhosis.

## Data Availability

The raw data of the laboratory values generated and analyzed during the current study are available from the corresponding author upon reasonable request.

## Supplementary Materials

The supporting information can be downloaded at: https://www.mdpi.com/article/10.3390/ijms241411351/s1.
