# Supplementary material for: Experimental Liver Cirrhosis Inhibits Restenosis after Balloon Angioplasty
Source: Int J Mol Sci. 2023 Jul 12;24(14):11351. doi: 10.3390/ijms241411351 (PMC10379020; doi:10.3390/ijms241411351)
Supplement: Supplementary file 1 [file ijms-24-11351-s001.zip › ijms-2486125-supplementary.pdf]

Supplementary Information

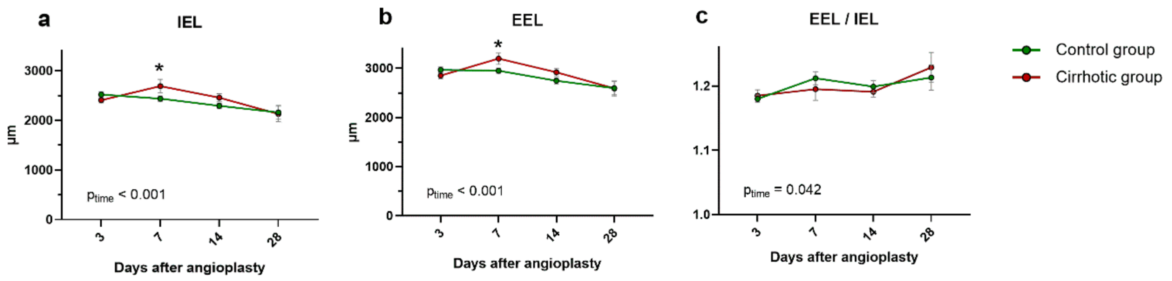

Figure S1. Results of planimetric analysis of histological cross-sectional areas of common carotid arteries in rats after bile duct ligation (cirrhotic group) versus sham surgery (control group) 3, 7, 14 and 28 days after vessel injury by balloon dilatation. Shown are the lengths of (**a**) the internal elastic lamina (IEL) and (**b**) the external elastic lamina (EEL), and (**c**) the ratio of EEL to IEL (EEL / IEL). Values are presented as mean ± standard errors of the mean (SEM). *p*-values (from generalized linear mixed model and pairwise group comparisons corrected for multiple comparisons): * $p < 0.05$ control vs. cirrhosis.

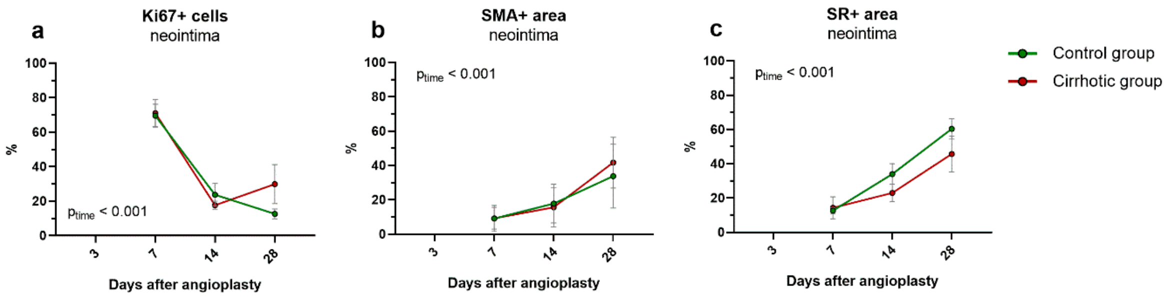

Figure S2. Immunohistology analysis of the neointima from the common carotid arteries of rats after bile duct ligation (cirrhotic group) versus sham surgery (control group) 3, 7, 14 and 28 days after balloon dilatation: (**a**) the percentage of ki67-positive (ki67+) cells to total cells, (**b**) the percentage of smooth muscle actin positive (SMA+) area, and (**c**) the percentage of Sirius Red-positive (SR+) area per cross-section. Values are presented as mean ± standard errors of the mean (SEM). *p*-values (from generalized linear mixed model and pairwise group comparisons corrected for multiple comparisons).

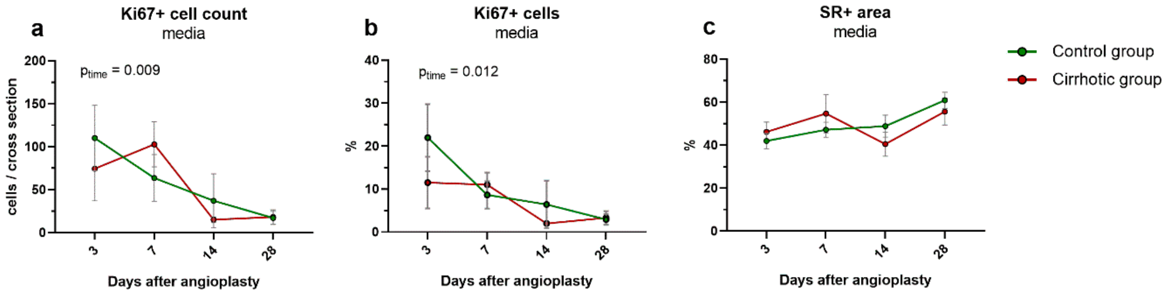

Figure S3. Immunohistology analysis of the media area from the common carotid arteries of rats after bile duct ligation (cirrhotic group) versus sham surgery (control group) 3, 7, 14 and 28 days after balloon dilatation: (**a**) the absolute number of ki67-positive (ki67+) cells, (**b**) the percentage of ki67-positive (ki67+) cells to total cells, and (**c**) the percentage of Sirius Red-positive (SR+) area per vessel cross-section. Values are presented as mean ± standard errors of the mean (SEM). *p*-values (from generalized linear mixed model and pairwise group comparisons corrected for multiple comparisons).

Table S1. Laboratory diagnostic: values for aspartate aminotransferase (AST), alkaline phosphatase (AP), bilirubin, albumin, creatinine and glucose in group comparison 3, 7, 14 and 28 days after balloon dilatation.

		Days following balloon dilatation			
		**3 days**	**7 days**	**14 days**	**28 days**
AST (U/L)	Control	77 ± 7	74 ± 4	76 ± 5	82 ± 12
	Cirrhotic	707 ± 121	320 ± 69	301 ± 31	349 ± 41
	p_{group}	*<0.001*	*<0.001*	*<0.001*	*<0.001*
AP (U/L)	Control	174 ± 19	192 ± 15	216 ± 16	255 ± 32
	Cirrhotic	426 ± 79	349 ± 95	273 ± 17	332 ± 21
	p_{group}	*0.003*	*0.003*	*0.03*	*0.03*
Bilirubin (µmol/L)	Control	3 ± 0.6	4 ± 0.9	4 ± 0.3	5 ± 0.3
	Cirrhotic	110 ± 13	135 ± 10	149 ± 16	152 ± 7
	p_{group}	*0.005*	*0.004*	*<0.001*	*<0.001*
Albumin (g/L)	Control	27 ± 0.5	27 ± 0.7	26 ± 0.9	28 ± 0.5
	Cirrhotic	25 ± 0.7	23 ± 1	25 ± 0.6	25 ± 0.6
	p_{group}	*0.007*	*<0.001*	*0.07*	*0.025*
Creatinine (µmol/L)	Control	45 ± 4	36 ± 3	31 ± 2	34 ± 3
	Cirrhotic	53 ± 7	43 ± 2	38 ± 2	44 ± 2
	p_{group}	*0.53*	*0.024*	*0.013*	*0.013*
Glucose (mg/dL)	Control	248 ± 15	255 ± 15	269 ± 6	257 ± 18
	Cirrhotic	148 ± 16	147 ± 12	169 ± 14	143 ± 8
	p_{group}	*0.002*	*0.001*	*0.001*	*<0.001*

Mean ± SEM.

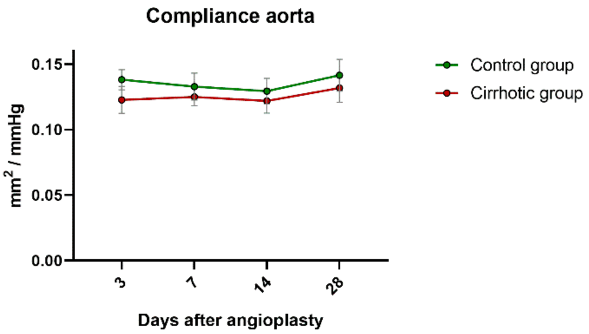

Figure S4. Aortic compliance 3, 7, 14 and 28 days after balloon dilatation in bile duct ligated (Cirrhotic group) and Sham-operated (Control group) rats. The aortic compliance [in $mm^2/mmHg$] was calculated using the difference between systolic and diastolic aortic cross-sectional area (Δ aortic cross-sectional area) from echographic images and the difference between systolic and diastolic pressure (ΔP) from conductance catheter measurements. The formula used was: compliance aorta = Δ aortic cross-sectional area / ΔP
